# Leading and Sustaining Curricular Change: Workshop Proceedings from the 2018 Sex and Gender Health Education Summit

**DOI:** 10.1089/jwh.2018.7387

**Published:** 2019-12-10

**Authors:** Kimberly Templeton, Leslie Halpern, Cynthia Jumper, Robert G. Carroll

**Affiliations:** ^1^ American Medical Women's Association, Orthopaedic Surgery, University of Kansas Medical Center, Kansas City, Kansas.; ^2^ Department of Surgery, University of Utah Health Sciences, Salt Lake City, Utah.; ^3^ University of Utah School of Dentistry, Salt Lake City, Utah.; ^4^ Health Policy and Special Health Initiatives, HSC, Lubbock, Texas.; ^5^ Medicine, Texas Tech University of Health Sciences, Lubbock, Texas.; ^6^ Brody School of Medicine at East Carolina University, Greenville, North Carolina.

**Keywords:** curricular change, education, interprofessional

## Abstract

The education of health science professionals must balance the incorporation of new and essential content against the current curriculum density. Scientific evidence documenting the impact of sex and gender on health outcomes establishes the need for incorporation of these topics into the health science curriculum. An interprofessional workshop was designed to provide participants with the knowledge and skills to effectively champion curricular change. Surveys before and after the workshop assessed the participants' perception of curriculum change. Introductory presentations addressed topics of organizational readiness and characteristics of change agents. This was followed by role-play activities in groups of 8 to 10, utilizing two scenarios. The first scenario involved a faculty champion advocating for change to the school curriculum leadership, and the second scenario involved the curriculum leadership advocating for change to the teaching faculty. After the role-play, participants shared the important points discovered by their groups, and the same information was collected by survey. After the workshop, 95% participants reported an increased ability to advocate for the inclusion of sex and gender topics in the curriculum. The most important aspect of the workshop was the providing of resources related to the teaching of sex and gender topics. We conclude that a workshop format balancing didactic information and role-playing scenarios is an effective tool for empowering faculty to introduce changes in health sciences curricula in areas that may be new to faculty or health science school leadership, such as the impact and role of sex and gender on health outcomes.

## Introduction

The curriculum in professional schools is dynamic, reflecting the influence of emerging health concerns, changes in the practice environment (many driven by technology), the nature and content of the institutional accreditation processes, and the requirement for individuals to be accredited as health care practitioners. Although increasing evidence indicates sex and gender differences in all health conditions, this knowledge is infrequently or inconsistently included in the education of health professionals.^1–5^ Framing education in this context could help to improve the health care provided to all patients, as well as to identify areas that need further study.^6^ The need for improvement in education in this area, as well as the potential impact on their future practices, has been identified by medical students.^7^

Within an institution, curriculum is determined by faculty, who are expected to remain informed about the innovations and evolutions shaping the teaching and learning environments.^8^ The curriculum is generally overseen by a committee of faculty and managed by a curriculum office of health professions education. Impetus for change—either addition or deletion from the curriculum—originates at many levels: concerns expressed by students or awareness of individual faculty members, course/block director, office of health professions education, or school leadership. Successful implementation of change requires at least tacit approval from each of these groups and can be facilitated by students who are supportive of these changes. With the pace at which new knowledge is becoming available, it is difficult to find time, or resources, within a curriculum to add new material.^9^ In addition, faculty may not have the required knowledge or background to teach these new fields of knowledge. This is no different when attempting to add sex and gender material to a curriculum.^2^ However, inclusion of the impacts of sex and gender is not accomplished by incorporation of a new course or clerkship: it is a fundamental shift in how health care professionals are taught and how they assess the literature and approach patients. As with other curricular changes, inclusion of this material is dependent on interest within a health professions school and/or interest or knowledge of the faculty.

A sex and gender in medical education summit was held in 2015, to initiate discussion on how to incorporate sex and gender education into medical school curricula.^10^ During this first summit, evidence was provided that described the impact of sex and gender in all health conditions, with initial discussions and recommendations about how to incorporate this knowledge into medical education. The primary recommendations from the initial summit^10^ were that sex and gender should be included in medical education, and that this content should be discussed throughout the curriculum, rather than in a separate course or elective. Barriers to inclusion of this material were identified, including the need to raise awareness among school leadership and the need to provide faculty development. To further discuss these barriers and practical tools to address them, The 2018 Sex & Gender Health Education Summit, “Advancing Curricula Through a Multiprofessional Lens,” was held in Salt Lake City, Utah, during April 8–10, 2018. This summit, which recognized the role interdisciplinary care plays in improving the health of all patients, was sponsored by the American Medical Women's Association (AMWA), the Laura W. Bush Institute for Women's Health at Texas Tech University Health Sciences Center, Mayo Clinic and the University of Utah Health.

An interprofessional workshop entitled “Leading and Sustaining Curricular Change” was held during the summit. The purpose of the workshop was several fold: evaluate the readiness of an organization for change, identify the characteristics of a change agent, identify how the inclusion of sex and gender-specific health topics can strengthen accreditation, and to provide scripting for faculty as they work with curriculum leaders in their respective schools to introduce sex and gender topics into the curriculum. The specific learning objectives of the workshop were to:

1. Understand the role of instructors, course/block directors, and curriculum committees in deterring curricular content and change.
2. Explain the impact of factors both internal and external to your institution (accreditation and clinical pressures) on shaping the curriculum.
3. Articulate benefits of including explicit sex and gender topics in the curriculum and harmful outcomes from failing to include these topics, as well as who or what would be impacted by benefits or harmful outcomes.

## Methods

Two hundred forty-six health care professionals including those from medicine, nursing, allied health, dentistry, and pharmacy and other health professions including occupational therapy and physical therapy attended the summit, with others participating *via* live-streaming, representing 23 countries. The largest contingent was physicians from the United States. As the road to change varies among institutions and programs, before the summit, participants were asked to contemplate a change that had been made in their curriculum and to identify what or who drove that change. They were also asked to determine whether that change had been successful and why or why not. At the workshop, the organizational readiness and characteristics of a change agent were accomplished *via* didactics. “Top-down” and “bottom-up” approaches to change were discussed, as well as rationales that could be used to persuade others when using either of those approaches. Scripting was accomplished *via* role-playing in small mixed professional groups of 8–10 professionals for those attending in person. After an hour of role-playing, each group, *via* a designated spokesperson, discussed their noteworthy ideas with the entire group of 246 attendees. These responses were recorded and collated into common themes.

The role-playing session consisted of two scenarios. The first scenario involved a workshop participant playing the role of faculty champion for sex and gender health presenting a request to either a school dean or the dean of curriculum to integrate this content into the curriculum. The person playing the role of the “school curriculum leadership” had been provided common obstacle responses that the workshop organizers had correlated from experience. The second scenario involved a workshop participant playing the role of the “School Dean” and holding a conversation with faculty, asking the faculty to integrate sex and gender materials into the curriculum.

The common obstacle responses provided by the workshop organizers included the following:

How much will it cost?

Isn't it taught in OB-GYN?

Who has the time to do a new curriculum?

Why now?

It is needed for accreditation?

Will the students be tested over it?

We don't want to get involved in politics.

We don't want to teach LGBTQ+.

We don't want to teach about abortions.

We already have Women's Health.

What do you want us to delete from the current curriculum?

After role-playing, each group in the audience participated in reviewing the important points that their group identified. In addition, evaluations consisted of a pre-and postworkshop survey. A total of 95 participants completed the preworkshop survey and 85 completed the postworkshop survey. Pretest versus Post-test was assessed by a chi square, *p* < 0.05.

## Results

*Post hoc* review of the comments generated in the group discussions noted that they fell into three main types of emphasis or strategies that would be useful to facilitate curricular change.

These three areas or emphasis and the related comments are as follows.

### Approach to preparation or framing of the discussion

State the request to add sex and gender into the curriculum as a “win/win” proposition

Use Kern's criteria^11^ for curriculum—six steps for curricular design

Be prepared

Expand within interprofessional education, avoid the “if/or,” rather use “and”

Identify where best (in the curriculum) to place it

Support with a needs assessment

Show how other schools are doing it

Emphasize that it is science based

Draw on extensive research that sex is a biological variable that affects individual health

Emphasize that every patient has a sex and a gender and they affect their health

Utilize good examples involving males and females (*i.e*., clinical scenarios or research)

Use the techniques of a motivational interview

Reframe existing goals/competencies)

Know the “pain” points for the stakeholders

Understanding is the key both for advocating effectively and ensuring the content are incorporated well

Be prepared with a plan for integration

Use analogies of other recent curricular innovations (*e.g*., Interdisciplinary Professional Education or IPE) to show integration is possible and advantageous

Plan for a pilot

Identify faculty champions

Include student input

### Link to outcomes (for the school, their graduate, and society)

Use inclusion of this curriculum as a recruiting tool

Cite the importance of NIH grants—some look favorably upon this science

Highlight student satisfaction component

Suggest opportunity to attract clinical and basic science researcher to the institution

Include in a strategic plan for the university/school

Include the opportunity to develop a reputation of the organization as an innovation leader

Show how our faculty/learners/students/residents/fellows will be better prepared to deliver excellent patient care if they understand the impact of sex and gender on health

Emphasize patient outcomes

Showcase precision medicine outcomes

Highlight forefront of educational innovation, medical practice, and patient care

### Acknowledge the roles of accrediting bodies and other outside forces

Provide evidence from “accrediting bodies”

Provide evidence from testing bodies

Incorporate into assessments

Provide evidence from national organizations—for example, the American Medical Association

Highlight LCME/AAMC/AMA/IOM(NAM)/ACGME recommendations—it is only a matter of time

There were no significant differences in the responses to questions on the post-test, compared with the pre-test, concerning curriculum leadership, such as who determines content and whom to approach to effect curricular change. More than half thought that the professional school faculty determined content, whereas only 5% thought that the dean of the school was in charge of determining content, with the remaining participants divided whether deans of education or licensing bodies filled this role. Similarly, in the pre- and post-test, most participants thought that curricular change should start with faculty or those within school leadership who are responsible for the curriculum, rather than approaching the dean of the school. In addition, participants recommended including medical students in new curricular initiatives.

There was also no change in responses on the post- compared with pretest on a question regarding where sex and gender content should be placed in the curriculum. The vast majority thought that sex and gender materials should be included throughout the undergraduate curriculum, rather than only within the preclinical or clinical years, with only a few commending the development of an elective in this area.

The relative impact of specific aspects of the workshop on participants' ability to advocate for the inclusion of sex and gender content in health professions' curricula was assessed in the post-test. Almost half (47%) of the participants noted the importance of the resources provided or noted during the summit. One-third noted the impact of peer learning, whereas 15% noted a positive impact from role-play, during which there were also opportunities for peer learning.

## Discussion

Curricular change, such as the inclusion of sex and gender content, can be challenging and may look different in each institution or department. This workshop sought to determine how best to include this content in a variety of health professions schools. Despite differences among schools, there was no change in the post-test compared with the pretest regarding opinions on who is responsible for leading and changing curricula, likely reflecting a group of participants who were involved in these roles at the various institutions represented. Participants also noted in the pre- and post-test that sex and gender content should be included throughout the health professions' education, rather than be isolated within the preclinical or clinical years or included within an elective. This lack of change as a result of the discussions at the meeting likely reflects the awareness of many participants that this recommendation has been made previously, such as a result of the sex and gender in medical education summit in 2015.

Role-play was utilized during this workshop as an innovative tool to identify and work through anticipated common objections to curricular change. Placing participants as both advocates and adversaries gave them an opportunity to reflect on the challenges, to anticipate roadblocks, and provide innovative solutions. In addition, issues of bias from faculty or administration were addressed to reflect on how this can alter the perception of sex and gender concepts being taught in the basic sciences and clinical settings. Despite the various health professions and schools represented at the workshop, several common themes arose during this role-play, including

1. how the incorporation of this curricula will align with the other initiatives/strategic plans for their institutions;
2. the need for faculty development curricula to teach their colleagues to “rethink” how they teach clinicians since many students/residents do not understand how these concepts impact the care of their patients; and
3. the impact of curricula requirements of accrediting bodies in their respective disciplines.

Participants noted that the inclusion of sex and gender education can lead to improved performance of the schools and their graduates. Linking these changes to the goals or missions of the schools can highlight to school leadership the importance of these changes, as well as how it aligns with their other initiatives. In addition, participants noted that these changes may be forced, at some point, by accrediting bodies. However, even without pressure from outside forces or bodies, many also noted the need to emphasize to those leading curricula that inclusion of sex and gender content will lead to schools producing health care professionals who will be better prepared to care for patients in their practices. All of these can be used as a part of a “bottom-up” approach to curricular change, arising from students and faculty. Inclusion of sex and gender content will require shifting or adding resources in terms of faculty development and support, and, potentially, development of new curricular resources and testing materials. Highlighting to those within institutions who are involved with distribution of needed resources that these changes can increase visibility for the schools, keep them on the cutting edge, and further their contribution to the welfare of society can be used as “win-win” propositions.

An important finding from this study is the high value that participants placed on teaching resources as tools to enhance their ability to advocate for sex and gender topics in the curriculum. All Workshop B attendees agreed that a universal sex and gender clinical scenario resource database can provide a springboard/foundation to enhance sex and gender-based concepts that can be translated within their health discipline. The development of a “toolkit” to enhance the ability to advocate for sex and gender topics in the curriculum was suggested by each faculty member so that an IPE collaborative can be applied to patient care: an ongoing precision model of sorts specific to medicine, dentistry, pharmacy, allied health sciences, and nursing. Such resources continue to be developed at places such as the Laura Bush Institute for Women's Health and the Office of Research on Women's Health at the NIH. Further development of these resources, as well as others, will make the inclusion of sex and gender materials more realistic and less daunting to faculty and curricula leaders.

An additional finding from this study was that role-play exercises and peer- learning strengthened the participants' confidence in advocating for inclusion of sex and gender differences in the curricula of their schools. This is critical because, as the participants noted, discussion of this type of curricular change can be challenging. Specific points of emphasis in the area of sex and gender differences were that “women's health” is not only gynecology and, as such, is not already included in most curricula. In addition, sex and gender health is not solely “women's health”—it is discussion of the differences in health and disease between men and women and among genders for all health conditions. In addition, sex and gender health does not describe differences based on the impact of gender alone—it is fundamentally based on the differences that occur as a result of chromosomal complement, i.e. (impacts of sex). Before conversations with faculty and curricular leaders, faculty champions need to recognize the broad expanse of this field. During the role-play, specific examples of the impacts of sex and gender on health and disease were provided, so that participants could use these during their discussions at their home institutions. In addition, these faculty champions also need to have developed clear plans on how and where to place this material in the curriculum, as well as already having developed support from other faculty and students. The role-play provided opportunities for participants to identify and practice specifics of these discussions.

## Limitations of this study

Although the objectives/goals of Workshop B were met with enthusiasm by all participants, the data collected must be carefully scrutinized for validity. Although the goal of this summit was to generate interdisciplinary dialogue, the majority of participants were from the field of medicine. Although the response rate appears to represent the majority of attendees, the responses may not accurately reflect the view of all the interprofessional participants within the workshop. The response rates by the other health disciplines may have, in addition, been influenced by a misunderstanding of the goals of Workshop B and its relevance to their specialty. In addition, since the majority of participants represented the discipline of medicine, selection bias must be considered, as medicine was the focus of the previous summit in 2015, and several participants attended the meetings in 2015 and the workshop that forms the focus of this discussion. In addition, those from other disciplines most likely were a self-selected group that either were already aware of or were interested in the area of sex- and gender-based health education.

Role-playing has the potential to be misinterpreted and can hinge upon previous knowledge and experience of the participants. This can significantly affect the concepts that are thought to be of significance by individual members of each group. In addition, not all summit participants actively participated in the role-play but, rather were observers of the interactions. Future studies should potentiate the power of this tool based upon each discipline's application of Workshop B's goals and objectives in their respective institutions.

## Summary

The 2018 Sex and Gender in Health Education Summit is the first of its kind to successfully utilize an IPE arena to provide the tools to support a paradigm shift for the inclusion of sex and gender curricula when training future health care providers. Sex and gender-based health educational curricula have the potential to be transparent and universally applied across all health disciplines. Workshop B entitled “Leading and Sustaining Curricula Change” provided a venue to flesh out common themes/challenges for curricular change by faculty participants across all health disciplines. This workshop provided several skill sets that allowed participants to work through the objections that are frequently cited as reasons to not proceed with this curricular change and to prepare them for conversations within their own schools that could be challenging. Follow-up surveys are being evaluated to see the progress by attendees in implementing some of the skills taught in Workshop B. The successful application of these resources and dynamic input of other clinical scenarios for the application of standardized sex and gender-based curriculum in an IPE format will form the groundwork/foundation for the third sex and gender-based summit in 2021.
